# A novel protein fusion partner, carbohydrate-binding module family 66, to enhance heterologous protein expression in *Escherichia coli*

**DOI:** 10.1186/s12934-021-01725-w

**Published:** 2021-12-28

**Authors:** Hyunjun Ko, Minsik Kang, Mi-Jin Kim, Jiyeon Yi, Jin Kang, Jung-Hoon Bae, Jung-Hoon Sohn, Bong Hyun Sung

**Affiliations:** 1 Synthetic Biology and Bioengineering Research Center, Korea Research Institute of Bioscience and Biotechnology (KRIBB), 125 Gwahak-ro, Yuseong-gu, Daejeon, 34141 Republic of Korea; 2 Department of Biosystems and Bioengineering, KRIBB School of Biotechnology, Korea University of Science and Technology (UST), 217 Gajeong-ro, Yuseong-gu, Daejeon, 34113 Republic of Korea

**Keywords:** Fusion tag, Carbohydrate-binding module, Soluble expression, Levan-agarose, *Escherichia coli*

## Abstract

**Background:**

Proteins with novel functions or advanced activities developed by various protein engineering techniques must have sufficient solubility to retain their bioactivity. However, inactive protein aggregates are frequently produced during heterologous protein expression in *Escherichia coli*. To prevent the formation of inclusion bodies, fusion tag technology has been commonly employed, owing to its good performance in soluble expression of target proteins, ease of application, and purification feasibility. Thus, researchers have continuously developed novel fusion tags to expand the expression capacity of high-value proteins in *E. coli*.

**Results:**

A novel fusion tag comprising carbohydrate-binding module 66 (CBM66) was developed for the soluble expression of heterologous proteins in *E. coli*. The target protein solubilization capacity of the CBM66 tag was verified using seven proteins that are poorly expressed or form inclusion bodies in *E. coli*: four human-derived signaling polypeptides and three microbial enzymes. Compared to native proteins, CBM66-fused proteins exhibited improved solubility and high production titer. The protein-solubilizing effect of the CBM66 tag was compared with that of two commercial tags, maltose-binding protein and glutathione-S-transferase, using poly(ethylene terephthalate) hydrolase (PETase) as a model protein; CBM66 fusion resulted in a 3.7-fold higher expression amount of soluble PETase (approximately 370 mg/L) compared to fusion with the other commercial tags. The intact PETase was purified from the fusion protein upon serial treatment with enterokinase and affinity chromatography using levan-agarose resin. The bioactivity of the three proteins assessed was maintained even when the CBM66 tag was fused.

**Conclusions:**

The use of the CBM66 tag to improve soluble protein expression facilitates the easy and economic production of high-value proteins in *E. coli*.

**Supplementary Information:**

The online version contains supplementary material available at 10.1186/s12934-021-01725-w.

## Background

*Escherichia coli* is a predominant workhorse in a wide range of biotechnological applications. It has been particularly employed as an efficient cell factory for the production of biomolecules, including high-value recombinant proteins. However, several heterologous proteins have been frequently produced as inactive protein aggregates called “inclusion bodies” during expression in *E. coli*. There are two approaches for dealing with inclusion bodies: counterpointing and avoiding. The former is the utilization of protein aggregates, with the advantage of yielding large amounts of protein. However, the inclusion body should be solubilized and refolded by the strong and large amount of detergents to recover the bioactivity of the target protein, which is a hurdle for scale-up production [1–3]. The latter comprises several strategies, including the optimization of culture conditions, host genome engineering, and the application of fusion tags to increase the solubility of target proteins [4].

Fusion tag technology is an attractive solution for inclusion body formation owing to its good performance in the soluble expression of target proteins, ease of application, and purification feasibility. To date, various peptides, proteins, and their derivatives have been developed and employed as fusion tags to enhance soluble protein expression and purification (Table 1). Maltose-binding protein (MBP) and glutathione-S-transferase (GST) tags are generally considered representative options for solubility enhancers, and hexa-histidine tag is the most widely used affinity tag [5]. Although various fusion tags have been developed and applied in recombinant protein production, it is impossible for a specific tag to carry an omnipotent solubilizing effect for various target proteins. Hence, researchers have developed novel fusion tags to expand the expression capacity of high-value proteins in *E. coli*.

**Table 1:** General fusion tags used in recombinant protein production

Tag	Protein	Origin	Size (kDa)	Purpose	References
MBP	Maltose-binding protein	*Escherichia coli*	43	B	[48]
GST	Glutathione-*S*-transferase	*Schistosoma japonicum*	27	B	[49]
Trx	Thioredoxin	*E. coli*	12	S	[50]
NusA	N-utilization substance	*E. coli*	54	S	[51]
SUMO	Small ubiquitin-modified	*Homo sapiens*	11	S	[52]
6 × His	Hexa-histidine	n/a	< 1	A	[53]
FLAG	FLAG-octapeptide	n/a	< 1	A	[54]
STREP	Streptavidin binding peptide	n/a	< 1	A	[55]
CBM66	Carbohydrate-binding module 66	*Bacillus subtilis*	18	B	This study

*n/a* not applicable, *B* bifunctional, *S* soluble expression, *A* affinity purification

Carbohydrate-binding modules (CBMs) are the components of carbohydrate-active enzymes with carbohydrate affinity [6]. Since the discovery of CBMs by Reese in 1950 [7], numerous CBMs have been identified and characterized [8]. In recombinant protein production, CBMs have been used as fusion partners for soluble expression and affinity purification with a combination of their carbohydrate ligands. The most studied system is the CBM3. Through the fusion of CBM3 from *Clostridium* sp., various proteins, including human heat-shock protein, antimicrobial peptides, and protein A, have been expressed in *E. coli* and purified using a cellulose matrix [9–12]. Another well-studied system, CBM2, has been shown to express proteins in various hosts such as *E. coli*, yeast, and mammalian cell lines [13, 14]. Furthermore, different target enzymes have been fused with CBM1, 9, and 30 and purified using a cellulose matrix [15–17]. Cuskin et al. recently reported a novel CBM family 66 (CBM66) from the exo-levanase of *Bacillus subtilis* (BsSacC). Using isothermal titration calorimetry and affinity gel electrophoresis, high binding affinity of CBM66 to fructans, particularly levan (β-2,6 fructan) was identified [18]. In addition, BsSacC was expressed well as a soluble enzyme in *E. coli* [19]; therefore, as with other CBMs, we considered the possibility of a soluble expression tag using CBM66.

Herein, we suggest the possibility of CBM66 as a novel fusion tag for the soluble expression of heterologous proteins in *E. coli*. The soluble expression capability of the tag was confirmed using various passenger proteins that are difficult to express in *E. coli*. The soluble expression-enhancing effect of the CBM66 tag was compared with that of commercial tags (MBP and GST) using a model protein. The novel protein fusion tag CBM66 can be used for the production of high-value proteins in the active form in *E. coli*.

## Results and discussion

### Construction of a plasmid for the expression of CBM66-fused proteins

**Fig. 1:**
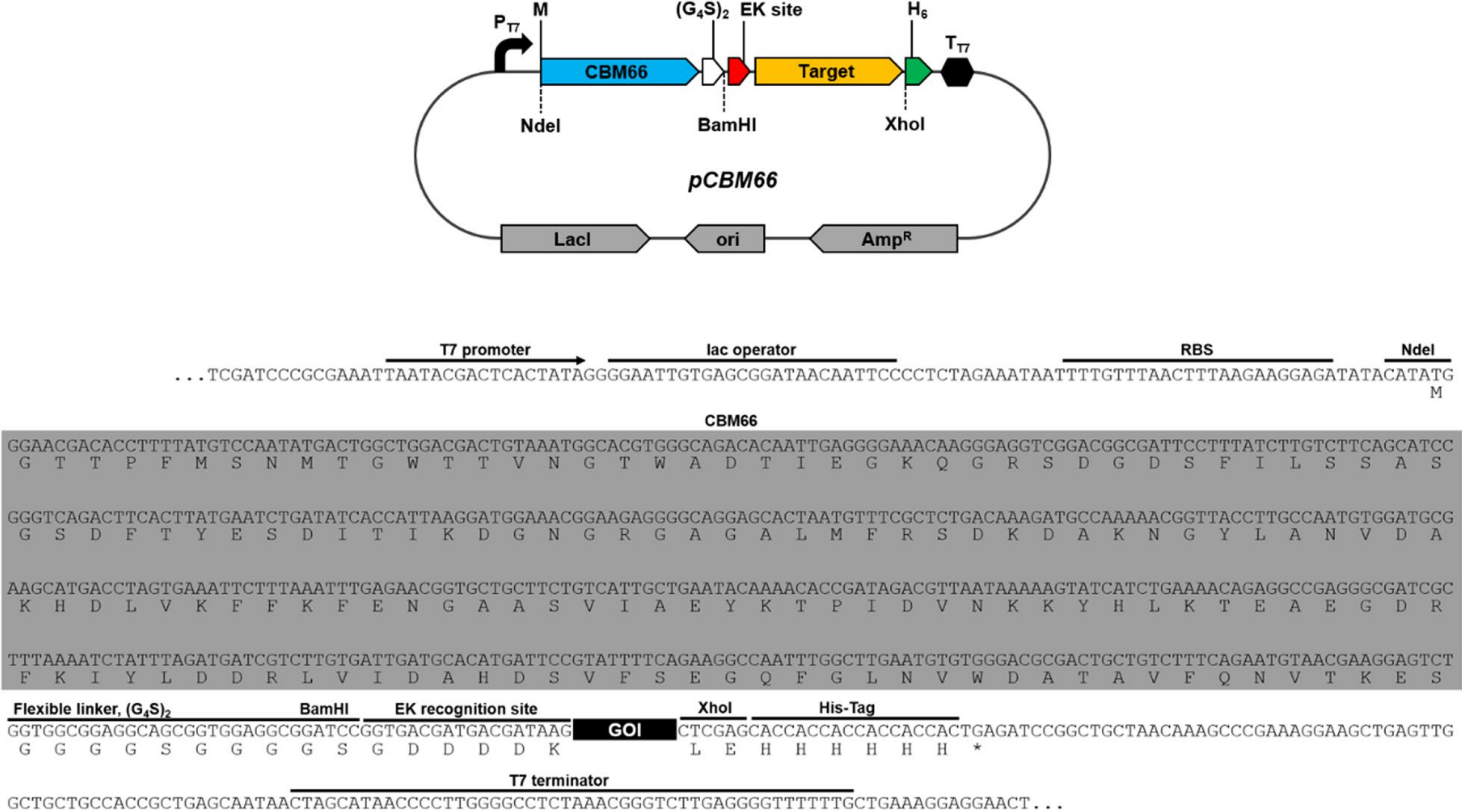
Map of the constructed protein expression plasmid harboring CBM66 fusion tag. The target protein was designed to be expressed with a CBM66 tag on the N-terminus and a His-tag on the C-terminus under the T7 promoter. The flexible linker between CBM66 and the passenger protein comprised a double repeat of four glycine and one serine [(G_4_S)_2_]. EK site indicates enterokinase recognition sequence (D_4_K)

To express target proteins fused with the CBM66 tag, the plasmid pCBM66 was constructed using the pET21b vector backbone. The target protein was designed to be expressed with a CBM66 tag on the N-terminus under the T7 promoter. A hexa-histidine-tag (his-tag) was attached to the C-terminus to purify and identify the expressed target proteins. In addition, a flexible linker domain, double repeats of four glycine and one serine [(G_4_S)_2_], and an enterokinase recognition site (D_4_K) were inserted between the tag and passenger protein for optional purification. To facilitate easy target gene cloning, recognition sites for two restriction enzymes (BamHI and XhoI) were inserted (Fig. 1). Although CBM66 naturally occurs in the C-terminus part of the levanase from *Bacillus subtilis* (BsSacC), we fused the CBM66 at the N-terminus of target proteins to obtain intact target proteins after enterokinase treatment, which cleaves after recognition sequence (D_4_K↓).

### Solubility enhancement of proteins by CBM66

**Fig. 2:**
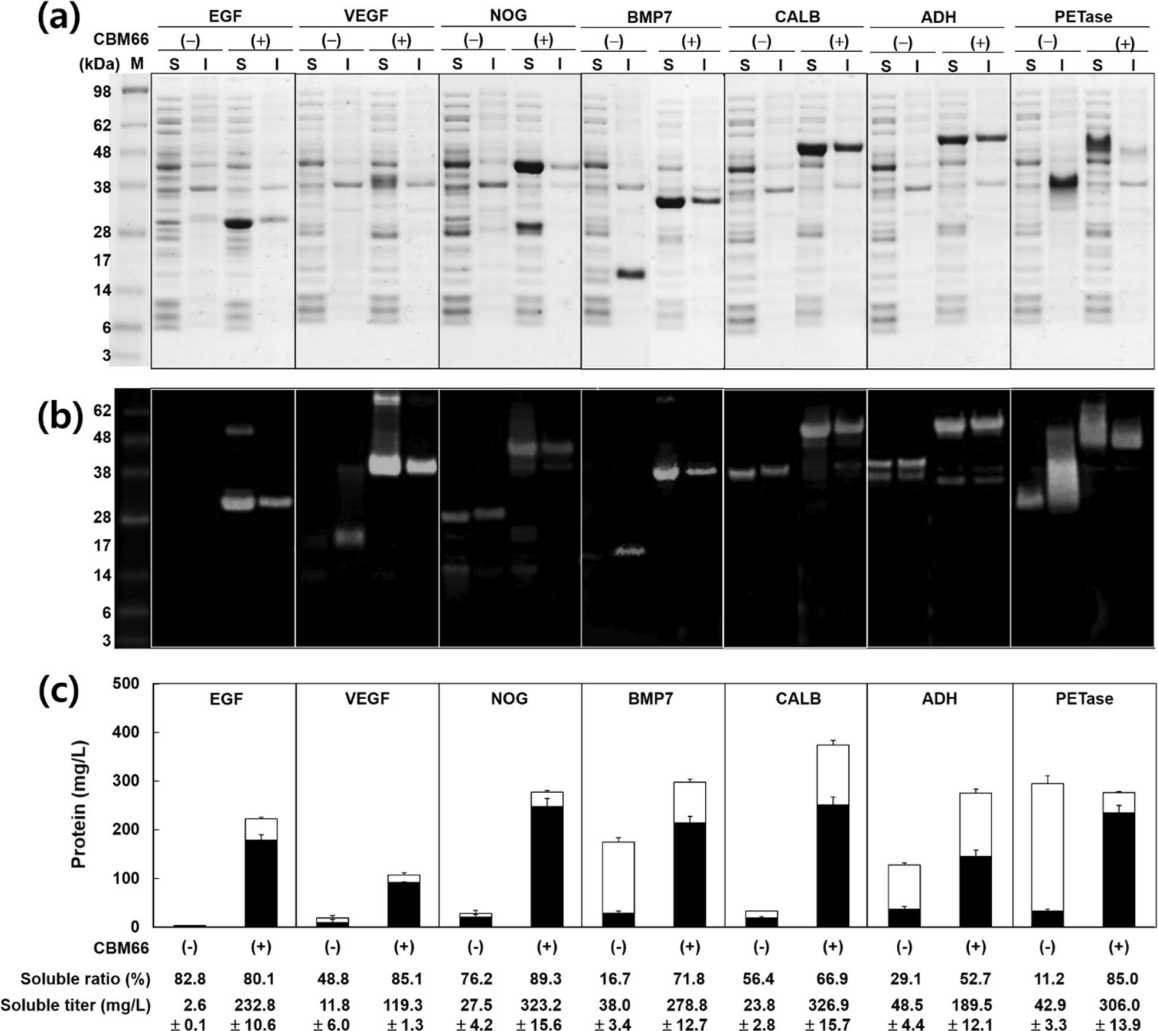
Soluble expression with the CBM66 tag. Expression profiles of seven target proteins analyzed using **a** SDS-PAGE and **b** western blotting. M, molecular marker; S, soluble protein; I, insoluble; ( −), without CBM66 tag; ( +), with CBM66 tag. **c** Quantification of expression profiles. The amount of soluble and insoluble proteins are represented as black and white bars, respectively. All images are representative of experiments performed in triplicate; all data are expressed as the mean ± standard deviation

To verify the soluble expression-enhancing effect of the CBM66 tag, we tested seven target proteins: four human-derived signaling polypeptides (epidermal growth factor [EGF], vascular endothelial growth factor isoform 165 [VEGF], noggin [NOG], and bone morphogenetic protein 7 [BMP7]) and three microbial enzymes (lipase B from *Candida antarctica* [CALB], alcohol dehydrogenase 1 from *Saccharomyces cerevisiae* [ADH1], and polyethylene terephthalate (PET) hydrolase from *Ideonella sakaiensis* [PETase]), which are difficult to express in *E. coli*. *E. coli* containing expression vectors was grown at 18 °C for 18 h after isopropyl β-d-1-thiogalactopyranoside (IPTG) induction, and when optical density at 600 nm (OD_600_) reached approximately 4.0 (Additional file 1: Fig. S1), the cells were harvested and protein expression was analyzed. As shown in Fig. 2, the solubility and productivity of the most proteins increased by the fusion of the CBM66 tag.

After fusion with CBM66, three human-derived signaling proteins, EGF, VEGF, and NOG, were detected in the soluble fraction, with titers of 233, 119, and 323 mg/L, respectively, whereas the recombinant proteins without the fusion tag were almost undetectable upon SDS-PAGE analysis after Coomassie blue staining. On SDS-PAGE, there were no proteins below 6 kDa. Therefore, we investigated whether EGF was not expressed or ran off the gel. When EGF purchased from Sigma-Aldrich (St. Louis, MO, USA) was loaded on the gel as a standard, it could be detected in SDS-PAGE (Additional file 1: Fig. S2). Based on this, it was confirmed that intact EGF was expressed to the extent that it could not be detected.

For the other human-derived protein, BMP7, protein solubility was inverted. A high amount of insoluble BMP7 (190 mg/L) was expressed without the CBM66 tag; however, CBM66-BMP7 was produced as a soluble protein (279 mg/L). For efficient expression of human signaling proteins, various fusion tags have been tested. As for EGF, thioredoxin (Trx) and GST tags have been employed; however, the tags were not effective in solubilizing EGF [20]. Viable soluble tags for EGF were developed based on small ubiquitin-related modifier (SUMO) tags. Su et al. first reported the feasible expression of SUMO-fused EGF, with 54.3 mg/L titer and 38.9% soluble expression ratio per total protein [21]. Subsequently, Ma et al. demonstrated an improved EGF production titer and the soluble expression ratio of 281 mg/L and 59.5%, respectively, by the fusion of Mxe GyrA intein with SUMO (Mxe-GyrA-SUMO) [22]. The production amount of CBM66-EGF (233 mg/L) was lower than that of the intein-SUMO-fused EGF. However, the soluble EGF expression ratio with CBM66 was 80.1%, and therefore, CBM66 is the most efficient fusion tag for soluble expression of EGF. For soluble expression of VEGF, various fusion tags, including MBP, GST, Trx, NusA, 6 × His, and two domains of human protein disulfide isomerase (PDI), were tested. From the tested tags, MBP, GST, NusA, and the two PDIs exhibited over 92% solubility of VEGF at 18 °C. For ease of purification, they selected MBP as the best option for VEGF production, and the production titer of MBP-VEGF was 64.4 mg/L [23]. In this study, the solubilizing effect of CBM66 for VEGF was determined to be 85.1%; however, the CBM66-VEGF production titer was 119 mg/L. NOG and BMP7 have been considered as proteins that are difficult to express in *E. coli*. Therefore, these proteins are expressed in mammalian cell lines [24, 25]. However, as mammalian cell culture requires elaborate culture conditions using expensive media, economic production techniques using microbial systems, must be established [26]. To the best of our knowledge, the CBM66 tag system was the first successful method for soluble expression of NOG and BMP7 in *E. coli.* In this study, although the CBM66 tag did not consistently exhibit the high titer or solubility of the tested human-derived proteins, the four human-derived target proteins were expressed with an average soluble ratio of over 81%, and the titer reached several hundreds of milligrams per liter, which is viable for high-yield production.

We also tested three microbial industrial enzymes (CALB, ADH1, and PETase) as passenger proteins to demonstrate the soluble expression capability of the CBM66 tag. CALB is the most extensively applied biocatalyst for the production of various oleochemicals [27]. However, *E. coli* has not been considered as an expression host for recombinant CALB because the protein is produced in negligible quantities or expressed as an inclusion body. To solubilize CALB, various biotechnological techniques such as codon optimization, mutagenesis, and co-expression with chaperones have been applied; however, the amount of soluble CALB produced was only several milligrams per liter [28–31]. Polycationic amino acid tags have recently been suggested for the soluble expression of CALB. Jung et al. constructed various polyamine tags comprising 10 consecutive homo basic amino acids (lysine, arginine, and histidine), and they selected the tag composed of 10 arginine residues as an optimal soluble tag for CALB by fusion at the carboxy terminus of the enzyme (CALB-10Arg) [32]. Similarly, Zhou et al. developed a more efficient soluble expression tag for CALB by the fusion of six histidine residues at the amino terminus and ten lysine residues at the carboxy terminus of CALB (6His-CALB-10Lys), and the production titer reached 100 mg/L [33]. In this study, by fusion with the CBM66 tag, the soluble CBM66-CALB exhibited 327 mg/L (Additional file 1: Fig. S3). ADH1 is a key enzyme involved in the metabolism of primary alcohols. Owing to its high stereoselectivity, it has been traditionally used in the production of drugs and chemicals [34]. ADH1 is a protein that is considered difficult to express in *E. coli*, and this was confirmed by the expression of insoluble ADH1 (71%) using the pET21b vector system; however, when ADH1 was fused to the CBM66 tag, the soluble ADH1 expression ratio increased to 53%, with 189 mg/L titer. PETase is a recently identified esterase from *I. sakaiensis* that hydrolyzes PET [35]. With increasing environmental issues due to the enormous usage and subsequent accumulation of petroleum-based plastics such as PET, biological degradation of the plastic by the enzyme has been suggested. Since its discovery, studies on enzymes have primarily focused on basic understanding, such as structural analysis of the mode of action, mutagenesis for the identification of crucial residues, and functional characterizations [36–40]. To utilize PETase in biological degradation of PET waste, a production system yielding sufficient amount of the enzyme has to be established. In this study, the level of CBM66-PETase was 360 mg/L, of which 306 mg/L (85%) was produced in the soluble form. However, 383 mg/L of intact PETase was produced, of which 11% (43 mg/L) was produced in the soluble form. Even in Rosetta-gami (DE3) strain, only about 50 mg/L of protein was produced in the soluble form (Additional file 1: Fig. S4).

Codon optimization is a predominant method for heterologous expression of recombinant proteins. However, in this study, we tested the soluble expression level of target proteins without codon optimization to avoid the misjudgment of solubilizing effect by CBM66 tag. For the seven proteins we tested, target proteins exhibited good expression levels in soluble form when fused with CBM66. However, it is difficult to predict whether CBM66 will function well for all proteins. In this case, better protein expression results can be obtained through codon optimization and the selection of various *E. coli* strains.

In this study, CBM66 tag was comprised the CBM66 domain following a repeat of four glycine and one serine (G_4_S)_2,_ and a specific protease recognition sequence (EK site, DDDDK). To verify whether the solubilizing effect of the CBM66 tag was derived from CBM66 not the linker and EK site, we constructed a pLE vector (Additional file 1: Fig. S5a), and tested expression titer using three target proteins (EGF, ADH, and PETase). ADH and PETase exhibited higher soluble expression with the LE tag than with canonical pET21b. However, the amount of obtained protein were < 50 mg/L (Additional file 1: Fig. S5b and c). Therefore, we concluded that the CMB66 tag was primarily responsible for the solubilizing effect.

The mechanism underlying solubility enhancement of fusion tags like CBMs has been investigated for decades; however, the correlation between tags and passenger proteins has not been elucidated. One dominant model for increased solubility of passenger proteins by CBMs is “chaperone-like quality.” Similar to MBP, various CBMs act as molecular chaperones that assist proper folding of attached proteins [16, 41, 42]. Despite the lack of understanding of CBMs, various CBMs have been utilized, and their applicability as fusion tags for heterologous expression and purification of various proteins has been proven [43, 44].

### Comparison of CBM66 with commercial tags

**Fig. 3:**
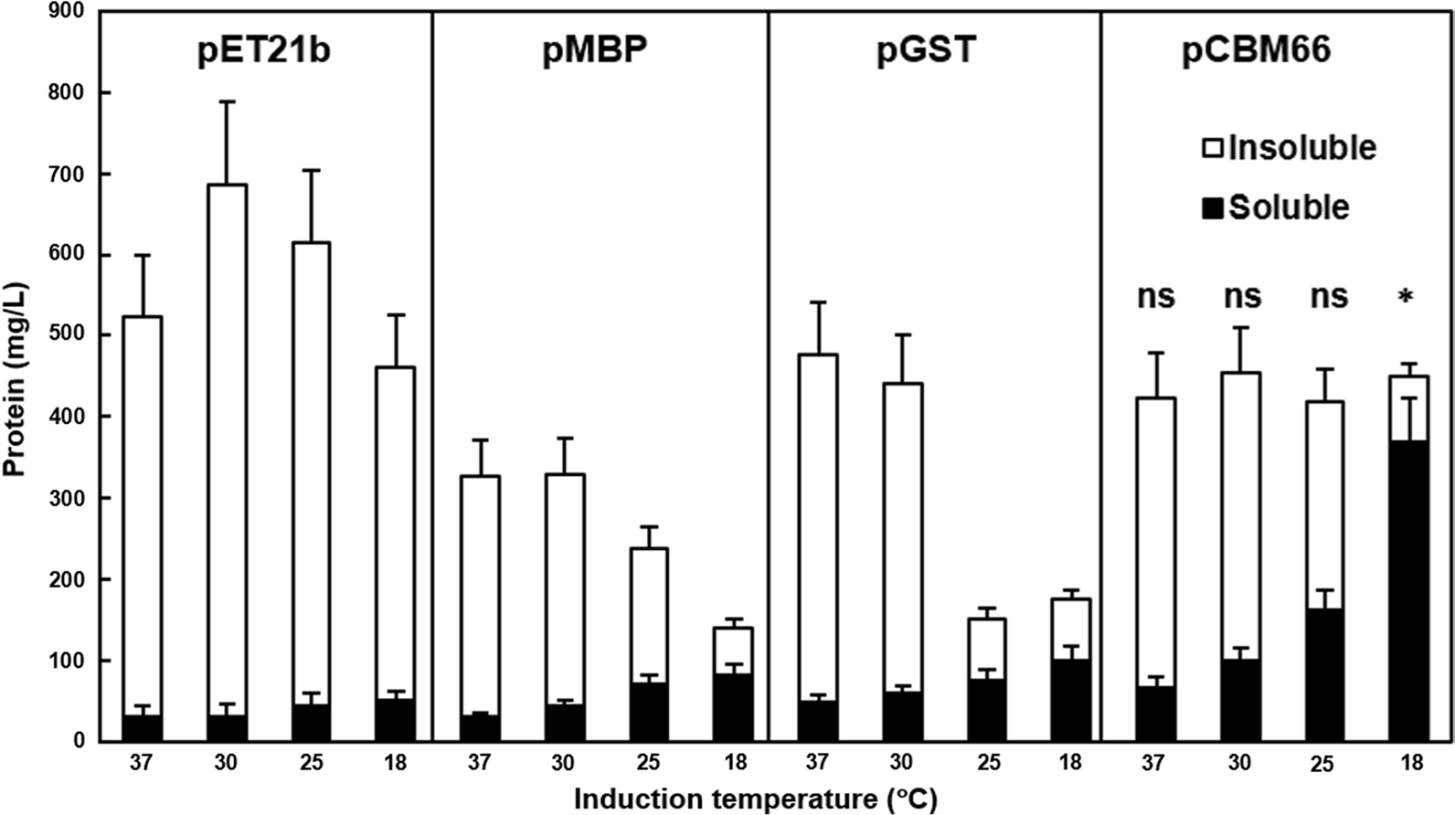
Expression of PETase with the CBM66, MBP, and GST fusion tags. Expression levels of PETase with different fusion tags at various induction temperatures. The amounts of soluble and insoluble proteins are represented as black and white bars, respectively. All images are representative of experiments performed in triplicate; all data are expressed as the mean ± standard deviation. The statistical significance of the data was analyzed by unpaired *t-test*. Values of *p* < 0.05 were considered to indicate statistically significant results. *p < 0.05, ns: not significant

The solubilizing efficacy of the CBM66 tag was compared with that of two commercial tags, MBP and GST. We selected PETase as a model protein because it has emerged as a countermeasure for the environmental issues of petro-derived plastics. As shown in Fig. 3, under induction temperatures of 37, 30, 25, and 18 °C, a high proportion of insoluble PETase was expressed in the absence of fusion tags. However, when a fusion tag (MBP, GST, or CBM66) was attached to the N-terminus of the passenger, the soluble expression level was increased at lower temperatures. Among them, the CBM66 tag exhibited the highest titer of soluble PETase (369 mg/L), and the soluble expression ratio was increased by over 82% at 18 °C. MBP and GST also exhibited increased soluble expression levels at 18 °C; however, the maximal concentration obtained by fusion with GST was approximately 100 mg/L. In addition, the total expression level with MBP and GST was decreased at lower temperatures; however, CBM66 maintained total protein production titer regardless of the induction temperature. Another advantage of the CBM66 tag is its smaller size (18 kDa) compared to the two commercial solubilizing tags (MBP, 43 kDa; GST, 26 kDa). Even if the soluble mass of the CBM66-tagged and MBP-tagged proteins were equivalent, the smaller CBM66 tag guarantees higher recovery yield of target proteins after tag removal procedure.

### Purification of intact protein from fusion protein using levan-agarose resin (LAR)

**Fig. 4:**
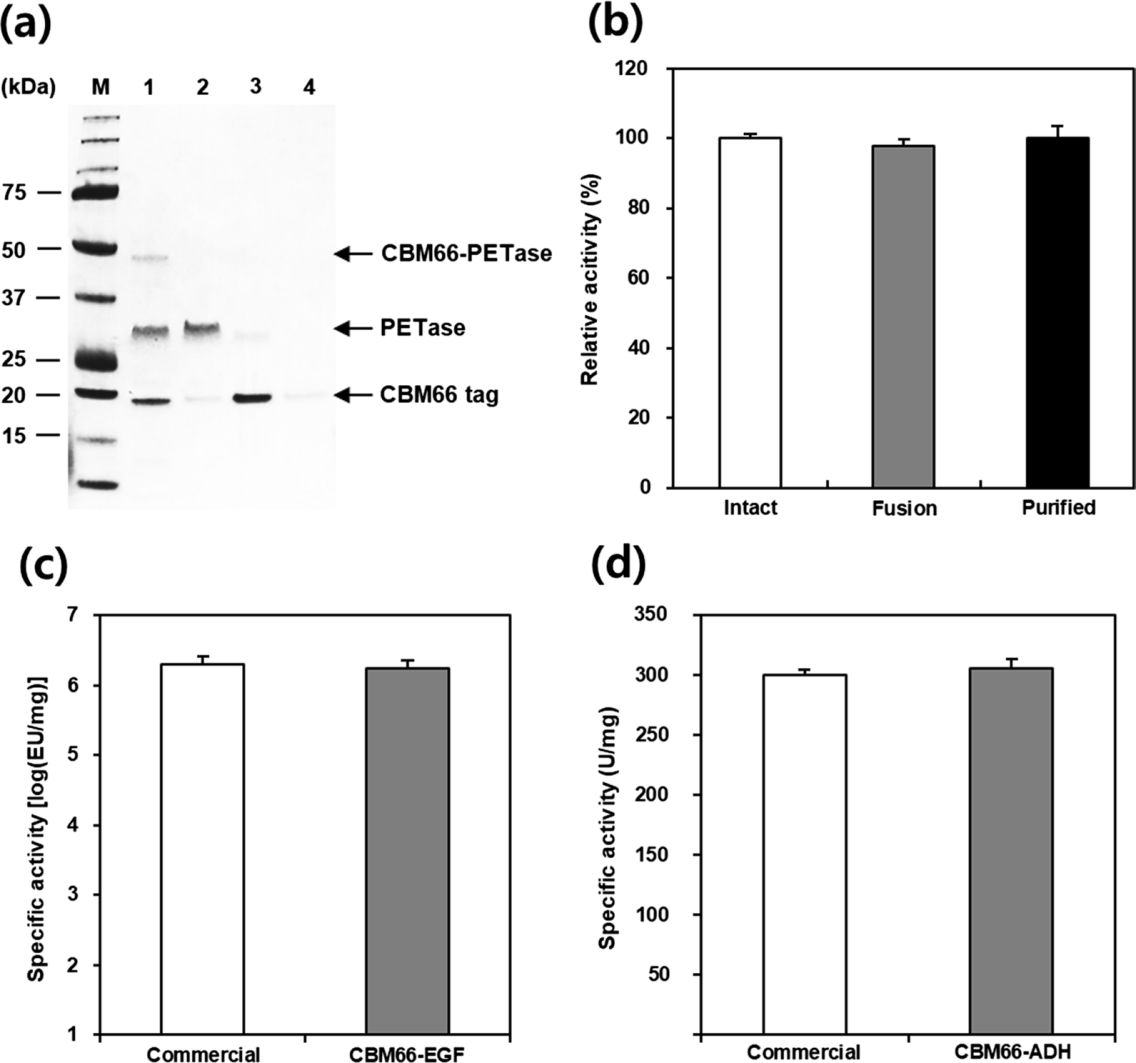
Purification of intact protein from the fusion product. **a** SDS-PAGE analysis of purification steps. The CBM66 tag and PETase cleaved by enterokinase (lane 1); PETase collected from flow through (lane 2). The CBM66 tag eluted with 50 mM and 100 mM sodium chloride solutions (lanes 3 and 4, respectively). **b** Bioactivity of PETase samples during the purification steps (intact, CBM66-fused, and purified PETase). Bioactivity of two CBM66-fused model proteins (EGF and ADH1) were compared with commercial enzymes on **c** and **d**, respectively. All images are representative of experiments performed in triplicate; all data are expressed as the mean ± standard deviation

To check whether CBM66-fused proteins could be purified using levan, a CBM66-binding carbohydrate, intact PETase cleaved from CBM66-PETase was purified using LAR. In detail, CBM66-PETase was purified by immobilized metal ion affinity chromatography (IMAC) using a His-tag on the C-terminus of the protein. Thereafter, the PETase and fusion tag were separated by enterokinase treatment. After cleavage, the reactant with 90.1% cleavage efficiency was directly loaded onto the prepared LAR. As shown in Fig. 4a, SDS-PAGE analysis revealed that the intact PETase did not interact with LAR and ran out as flow through. In contrast, the CBM66 tag was bound to LAR and was fully eluted using 100 mM NaCl. From the quantitative analysis of each fraction, the recovery yield of cleaved intact PETase was 88.6%.

Most CBMs identified thus far are cellulose-binding modules. Cellulose is a good purification matrix owing to its stability, safety, and cost-effectiveness. However, the binding affinity between CBMs and cellulose is extremely high, and thus, strong protein denaturation reagents such as urea and guanidine hydrochloride are required for the detachment of CBMs from cellulose followed by dialysis [43]. Based on the binding nature of CBM66, we prepared LAR as the purification matrix, and the target protein was easily eluted from the matrix using sodium chloride, which is a favorable reagent for protein studies. The purification system used in this study has not yet been fully established, which limits the precise specification of the levan matrix. Therefore, to improve CBM66-based soluble expression and levan-based purification systems, it is necessary to determine the precise binding capability, physical and chemical stability, and other characteristics of the levan matrix.

### Bioactivity of CBM66-fused proteins

To analyze the effect of CBM66 tag on the bioactivity of the CBM66-fused protein, the relative activities of CBM66-PETase, -EGF, and -ADH1 were compared with those of the corresponding intact proteins. The intact PETase was obtained from a large volume culture of *E. coli* BL21 (DE3)/pET21b vector system, followed by IMAC purification. PETase activity was calculated as the hydrolysis activity of bis(2-hydroxyethyl) terephthalate (BHET). The biological activities of CBM66-PETase and detagged PETase were 97.9 ± 1.3% and 100.1 ± 1.4% of the biological activity of the commercial PETase, respectively (Fig. 4b). Specific activity of CBM66-EGF was 1.8 × 10^6^ EU/mg, which corresponded to 97.6 ± 2.5% of that of commercial EGF (Fig. 4c). For ADH, both commercial and CBM66-fused ADH exhibited similar specific activity (300.1 ± 4.9 and 305.4 ± 8.2 U/mg, respectively) within the margin of error (Fig. 4d). Overall, all tested fusion proteins were found to exhibit biological activities similar to those of the corresponding intact proteins.

## Conclusions

In this study, we developed a novel protein fusion tag using CBM66. The capacity of CBM66 to solubilize target proteins was investigated using seven target proteins, and the CBM66 tag exhibited increased soluble protein expression and total expression levels. The solubilizing effect of CBM66 was compared with that of MBP and GST using PETase as a model protein. CBM66 exhibited the highest soluble protein expression compared to other commercial tags. Furthermore, the possibility of using CBM66 as a protein purification tag was applied to intact PETase purification through serial treatment with a specific protease (enterokinase) and affinity chromatography via LAR. To date, there have been numerous studies aiming to overcome inclusion body formation during recombinant protein production in *E. coli*; however, there is no universal solution in biological systems. Thus, we expect that the CBM66 tag can be an alternative protein fusion tag for efficient production of high-value proteins in *E. coli*.

## Methods

### Strains, chemicals, and media

*Escherichia coli* DH5α [F^−^ *lac*ZΔM15 *hsd*R17(r- m-) *gyr*A36] and BL21(DE3) [F^−^ ampT *dcm hsd*Sβ (rβ- mβ-) *galλ* (DE3)] were employed for genetic manipulation and expression of the target proteins, respectively. Q5 DNA polymerase, enterokinase, and restriction endonucleases were purchased from New England Biolabs (Ipswich, MA, USA). The In-Fusion HD cloning kit was purchased from Clontech Laboratories (Mountain View, CA, USA). The prepared DNA was purified using Wizard SV Gel and the PCR Clean-Up system (Promega, Madison, WI, USA). The Quick-DNA Miniprep Kit was purchased from Zymo Research (Irvine, CA, USA). Levan was purchased from Real Biotech (Gongju, Korea). All other chemicals were purchased from Sigma-Aldrich. The *E. coli* transformants were cultured in lysogeny broth (LB) containing 100 μg/mL ampicillin.

### Construction of recombinant vector harboring the CBM66 tag

The protein expression vector pCBM66 was designed by the insertion of a flexible linker domain, (G_4_S)_2_, and enterokinase cleavage site between the tag and passenger protein for optional purification. The recombinant vector was constructed on the pET21b vector backbone. The CBM66 fragment (residues 514–677 of BsSacC) was prepared from the genomic DNA of *B. subtilis* [18]. The strain was obtained from the Korean Collection for Type Cultures (KCTC#2217, ATCC33234). To amplify the fragment, primers (F1 and R1) were designed based on the NCBI database (NP_390581.1), including NdeI and XhoI recognition sites, and synthesized by Genotech (Daejeon, Korea). The linker was added to the carboxyl terminus of CBM66 by PCR using primers F2 and R2, and the enterokinase recognition sequence was added to the amino terminus of the target protein using specific primers F3 and R3. The fragments were cloned into the NdeI/XhoI double-digested pET21b vector using the In-Fusion HD cloning kit, and the recombinant vector was introduced into BL21(DE3). To compare the soluble expression efficacy of CBM66 with commercial tags, the CBM66 region of the vector was replaced by MBP and GST. Each gene was prepared from pMAL-p5X (NEB) and pGEX-4T1 (GE Healthcare, Little Chalfont, UK) vectors, respectively, using PCR with the F4/R4 and F5/R5 primer sets, respectively. To construct a vector, pLE containing only a linker [(G_4_S)_2_] and an enterokinase recognition site (DDDDK) was used. Each gene was amplified using F6 and R3 primers. The amplicon was ligated with the linearized pET21b vector by NdeI/XhoI (Additional file 1: Fig. S5). Cloning was performed as described previously. The genes of seven passenger proteins from the NCBI database [EGF (GenBank: AAS83395.1), VEGF (GenBank: AAL27630.1), NOG (PDB: 1M4U_A), BMP7 (GenBank: AIL24690.1), CALB (PBD: 1LBT_B), ADH1 (PDB: 4W6Z_A), and PETase (PDB: 6EQD_A)] were synthesized by Bioneer Corp. (Daejeon, Korea). Nucleotide sequences of the primers are listed in Table 2.

**Table 2:** Primers used in this study

Amplicon	Primer	Sequence (5’ to 3’)
CBM66	F1	AGAAGGAGATATACATATGGGAACGACACCT
	R1	GTAACGAAGGAGTCTCTCGAGCACCACCAC
CBM66-L^a^	F2	GTAACGAAGGAGTCTGGTGGCGGAGGCAGCGGTGGAGGCGGATCC
	R2	ATCGTCATCGTCACCGGATCCGCCTCCACCGCTGCCTCCGCCACC
CBM66-L-EK^b^	F3	GGTGGAGGCGGATCCGGTGACGATGACGATAAGXXXXXXXXXXXXXXX
	R3	XXXXXXXXXXXXXXXTCAGTGGTGGTGGTGGTGGTGCTCGAG
pMAL-L-EK	F4	GGAGATATACATATGAAAATAAAAACAGGT
	R4	GCTGCCTCCGCCACCAGTCTGCGCGTCTTT
pGST-L-EK	F5	GGAGATATACATATGTCCCCTATACTAGGT
	R5	GCTGCCTCCGCCACCTTTTGGAGGATGGTC
pLE	F6	AGAAGGAGATATACATATGGGTGGCGGAGGC

^a^L, flexible linker domain [double repeat of four glycine and one serine, (G_4_S)_2_]

^b^EK, enterokinase cleavage site

X, nucleotides of target genes

### Protein expression analysis

*E. coli* BL21(DE3) was transformed with the plasmids constructed for the expression of the passenger proteins, and each transformant was cultured in a 250 mL Erlenmeyer flask containing 50 mL LB at 37 °C and 180 rpm until the OD_600_ was 0.4–0.6. To induce protein expression, 0.1 mM IPTG was added to the culture medium and incubated at 18 °C for 18 h. After cultivation, the cells were harvested by centrifugation at 8000 × *g* for 5 min and reconstituted in 10 mL of 25 mM Tris–HCl buffer (pH 7.5). The cells were disrupted by ultrasonication for 5 min with a 3 s pulse interval on ice. The cell lysate was centrifuged at 15,000 × *g* for 15 min, and the supernatant and pellet were collected. The supernatant was used as a soluble protein sample, and insoluble protein was obtained from the pellet using an equal volume of xTractor buffer following the manufacturer’s instructions (Takara Bio, Shiga, Japan). To compare the expression ratio of soluble and insoluble portions, 10 μL of prepared samples were analyzed by SDS-PAGE. Proteins expressed with His-tag were purified and desalted using a Profinia protein purification system employing 5/50 mL Bio-scale mini Profinia affinity/desalting cartridges following the manufacturer’s instructions (Bio-Rad, Hercules, CA, USA). Quantitative analysis was performed by a densitometry assay using Image Studio Lite (Licor Inc., Lincoln, NE, USA). Purified protein samples were quantified using the Pierce BCA protein assay kit following the manufacturer’s instructions (Thermo Fisher Scientific, Rockford, IL, USA), and the known concentrations of the proteins were loaded on SDS-PAGE to set the standard curves. A comparison analysis of the soluble expression efficacy of CBM66 with two commercial tags (MBP and GST) was performed as described above at various induction temperatures. The temperature was set from 37 °C to 18 °C.

### Preparation of LAR

LAR was prepared by following a homemade amylose–agarose column preparation method [45]. Briefly, 25 mL Sepharose 6B (GE Healthcare) was sequentially washed with water and 1 M sodium carbonate solution, and the resin was reconstituted in 25 mL of 1 M sodium carbonate and 5 mL of vinyl sulfonate for 70 min. The resin was then washed with water and resuspended in 25 mL of 1 M sodium carbonate containing 1.25 g of levan for 16 h with gentle stirring. The resin was washed with water, then with 0.9% NaCl, and again with water. The prepared resin was stored in 20% ethanol solution at 4 °C, and the binding yield of levan onto the agarose was measured via high performance liquid chromatography (HPLC) [46].

### Removal of the CBM66 tag and purification of target protein

Before the removal of the CBM66 tag from the fusion protein by enterokinase, the fusion protein was partially purified using the Profinia protein purification system as described above. The purified fusion protein was reconstituted in cleavage buffer (20 mM Tris–HCl, 50 mM NaCl, 2 mM CaCl_2_, pH 8.0) using an Amicon centrifugal filter cartridge (Merck, Kenilworth, NJ, USA). One unit of enterokinase per 25 µg of the fusion protein was added to the reaction mixture and incubated at 25 °C for 16 h. After the reaction, the reactant was loaded directly into the open column containing the prepared LAR. Intact target protein was collected from the flow through, and the fusion tag was eluted with 50 mM and 100 mM sodium chloride. Each fraction was analyzed using SDS-PAGE.

### Bioactivity analysis

PETase activity was verified based on previous reports using BHET as a substrate [35, 47]. In detail, 500 μL aliquots of 100 nM enzyme samples were added to 500 μL of 20 mM Tris–HCl buffer (pH 7.5) containing 2 mM BHET and 1% DMSO. The reaction was performed at 30 °C for 30 min and halted by heating at 85 °C for 15 min. The activity was calculated by quantitative analysis of degraded BHET detected using HPLC. The 1100 series HPLC and ZORBAX Eclipse XDB-C18 column (Agilent, Santa Clara, CA, USA) were used for the quantitative analysis of BHET. Compounds were detected at 260 nm wavelength. Mobile phase A (0.1% formic acid) and B (acetonitrile) were used at a flow rate of 0.8 by gradually increasing B from 5 to 65% over 25 min.

EGF activity was verified by cell proliferation analysis. Human keratinocyte HaCaT cells were cultured in Dulbecco’s modified Eagle’s medium (DMEM) supplemented with 10 U/mL of penicillin–streptomycin and 10% fetal bovine serum (FBS) (DMEM + / +) at 37 °C with 5% carbon dioxide in a 96-well microplate. The initial inoculum was adjusted to 2 × 10^4^ cells/mL in the prepared media and incubated for 48 h. After 48 h, the medium was replaced with fresh DMEM + / − (without FBS) containing intact and CBM66-fused EGF at a final concentration of 1 nM and incubated for 48 h. The colorimetric assay of cell proliferation was performed using a water-soluble tetrazolium salt (EZ-Cytox, Daeillab Service, Seoul, Korea). One-tenth volume of EZ-Cytox was added to each culture and incubated for 5 h. Cell viability was analyzed by measuring the OD_540_.

The bioactivity of ADH1 was confirmed by the formation of acetaldehyde from ethanol. An aliquot of 5 nM of each protein sample was added to 1 mL of reaction mixture containing 50 mM ethanol and 25 mM NAD^+^. The mixture was incubated at 30 °C for 2 h. After incubation, 50 μL of 3 M HCl was added to the mixture to halt the reaction. The amount of acetaldehyde produced was quantified using HPLC analysis. The Animex HPX-87H column (Bio-Rad) was equipped with 1100 series HPLC (Agilent), and HPLC grade water containing 5 mM sulfuric acid was used as the mobile phase at a flow rate of 0.6.

One unit of EGF (EU) was defined as the amount peptide that generates 50% cell proliferation effect via MTT assay described above. One unit of ADH was defined as the amount of enzyme produced by 1 μM acetaldehyde from ethanol per minute. Standard EGF and ADH1 were purchased from Sigma–Aldrich.

## Supplementary Information

**Additional file 1: Figure S1**. Cell growth curve of *E. coli* BL21(DE3) containing PETase expression vectors. **Figure S2**. SDS-PAGE analysis of commercial EGF. **Figure S3**. Quantification of CBM-fused CalB lipase. **Figure S4**. Expression analysis of PETase in *E. coli* Rosetta-gami (DE3). **Figure S5**. Soluble expression effect of a linker and a peptidase domain in pCBM66.

## Data Availability

All data generated or analyzed during this study are included in this published article.

## Abbreviations

CBM66: Carbohydrate-binding module family 66
LAR: Levan-agarose resin
MBP: Maltose-binding protein
GST: Glutathione S-transferase
HPLC: High performance liquid chromatography
BHET: Bis (2-Hydroxyethyl terephthalate)
EGF: Epidermal growth factor
DMEM: Dulbecco's modified Eagle's media
FBS: Fetal bovine serum
ADH1: Alcohol dehydrogenase 1
PETase: Poly(ethylene terephthalate) hydrolase
IMAC: Immobilized metal ion affinity chromatography
