# Supplementary material for: A novel protein fusion partner, carbohydrate-binding module family 66, to enhance heterologous protein expression in Escherichia coli
Source: Microb Cell Fact. 2021 Dec 28;20:232. doi: 10.1186/s12934-021-01725-w (PMC8715580; doi:10.1186/s12934-021-01725-w)

**Supplementary Information**

**A novel protein fusion partner, carbohydrate-binding module family 66, to enhance heterologous protein expression in *Escherichia coli***

Hyunjun Ko^1^, Minsik Kang^1,2^, Mi-Jin Kim^1^, Jiyeon Yi^1^, Jin Kang^1,2^, Jung-Hoon Bae^1^, Jung-Hoon sohn^1,2,*^, and Bong Hyun Sung^1,2,*^

^1^Synthetic Biology and Bioengineering Research Center, Korea Research Institute of Bioscience and Biotechnology (KRIBB), 125 Gwahak-ro, Yuseong-gu, Daejeon 34141, Republic of Korea

^2^Department of Biosystems and Bioengineering, KRIBB School of Biotechnology, Korea University of Science and Technology (UST), 217 Gajeong-ro, Yuseong-gu, Daejeon 34113, Republic of Korea.

Hyunjun Ko and Minsik Kang contributed equally to this work.

Correspondence should be addressed to B.H. Sung ([bhsung@kribb.re.kr](mailto:bhsung@kribb.re.kr)) and J-H. Sohn ([sohn4090@kribb.re.kr](mailto:sohn4090@kribb.re.kr)).

**Supplementary Figure 1.** Cell growth curve of *E. coli* BL21(DE3) containing PETase expression vectors.

**Supplementary Figure 2.** SDS-PAGE analysis of commercial EGF.

**Supplementary Figure 3.** Quantification of CBM-fused CalB lipase.

**Supplementary Figure 4.** Expression analysis of PETase in E. coli Rosetta-gami (DE3).

**Supplementary Figure 5.** Soluble expression effect of a linker and a peptidase domain in pCBM66.

**Supplementary Figure 1.** Growth curve of *E. coli* BL21(DE3) containing PETase expression vectors. Cell growth curves under the pre-culture at 37 °C for 2 h following expression-culture at 18 °C (IPTG induction) for 18 h were measured by optical density at 600 nm.

**
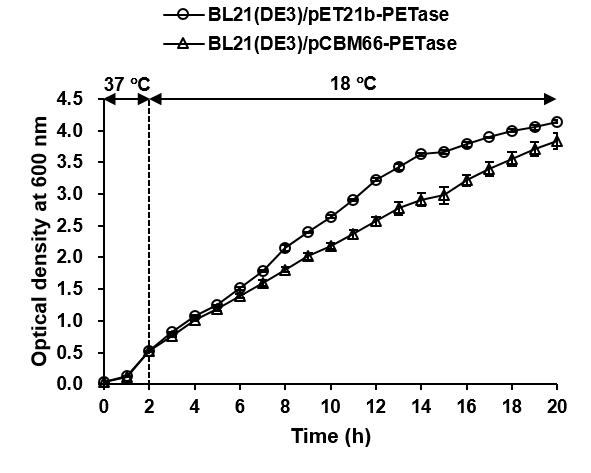
**

**Supplementary Figure 2.** SDS-PAGE analysis of commercial EGF. M, molecular marker; S, standard EGF purchased from SIGMA-ALDRICH; E, EGF expressed in this study; CE, CBM66-EGF expressed in this study.


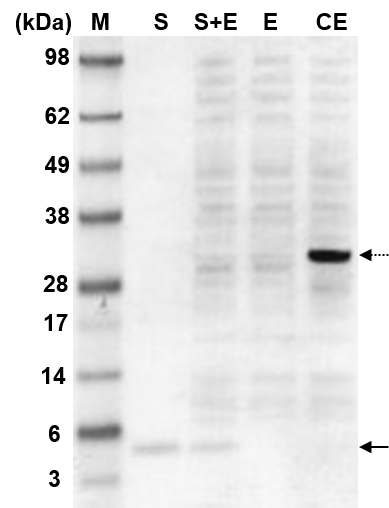


**Supplementary Figure 3.** Quantification of CBM-fused CalB lipase. Purified protein was quantified using the BCA assay and the known concentrations of the proteins were loaded on SDS-PAGE (a), quantitative analysis was performed by a densitometry assay based on the standard curves (b). M, molecular marker; S, protein sample.


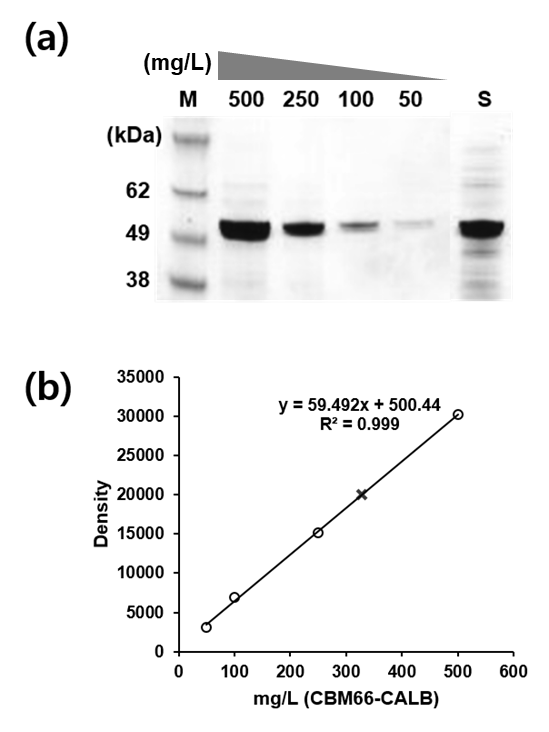


**Supplementary Figure 4.** Expression analysis of PETase in the *E. coli* Rosetta-gami (DE3). PETase expressed in Rosetta-gami (DE3) stain under the same condition in this study without codon optimization, and the expression was analyzed by SDS-PAGE (a) and western blotting (b). M, molecular marker; S, soluble protein; I, insoluble protein.


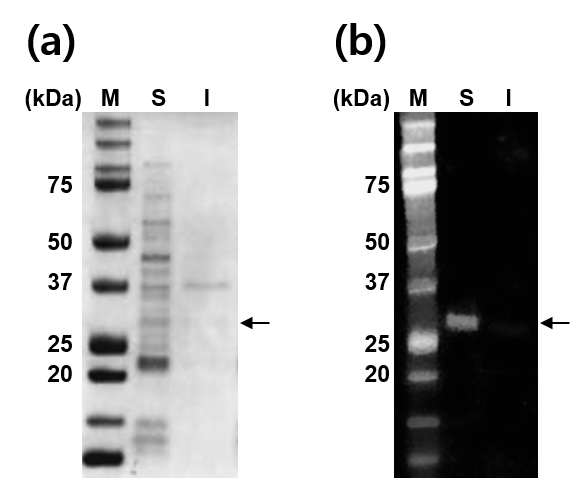


**Supplementary Figure 5.** Soluble expression Effect of a linker and a peptidase domain in pCBM66. Construction of pLE vector containing linker [(G_4_S)_2_] and enterokinase recognition site (GDDDDK) from the CBM66 tag (a). Soluble expression of EGF, ADH, and PETase was analyzed by SDS-PAGE (b) and western blotting (c). M, molecular marker; S, soluble protein; I, insoluble protein. ADH was indicated as solid arrow and PETase was indicated as dotted arrow.


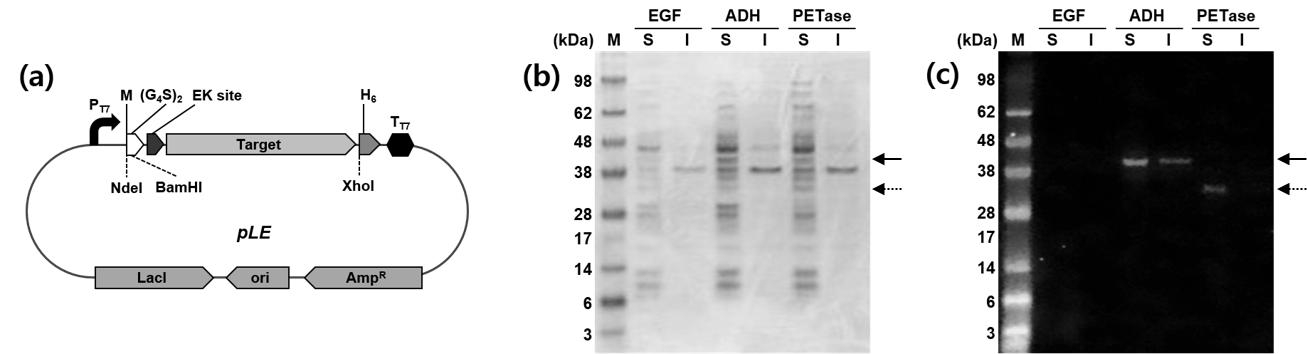

Supplement: Supplementary file 1 — Additional file 1: Figure S1. Cell growth curve of E. coli BL21(DE3) containing PETase expression vectors. Figure S2. SDS-PAGE analysis of commercial EGF. Figure S3. Quantification of CBM-fused CalB lipase. Figure S4. Expression analysis of PETase in E. coli Rosetta-gami (DE3). Figure S5. Soluble expression effect of a linker and a peptidase domain in pCBM66. [file 12934_2021_1725_MOESM1_ESM.docx]
